# Associations Between the Periodontal Disease in Women Before Menopause and Menstrual Cycle Irregularity: The 2010–2012 Korea National Health and Nutrition Examination Survey

**DOI:** 10.1097/MD.0000000000002791

**Published:** 2016-02-12

**Authors:** Kyungdo Han, Youngkyung Ko, Yong Gyu Park, Jun-Beom Park

**Affiliations:** From the Department of Biostatistics (KH, YGP); and Department of Periodontics, College of Medicine, The Catholic University of Korea (YK, J-BP), Seoul, Republic of Korea.

## Abstract

The association between menstrual cycle irregularities and system disease has been evaluated in previous studies. However, the association between periodontal disease and menstrual cycle irregularity has not been fully investigated. The study aimed to evaluate the relationship between periodontal disease and tooth loss in women before menopause and menstrual cycle irregularity using nationally representative data.

This study performed a cross-sectional analysis and used hierarchical multivariable logistic regression analysis models. Data from Korean National Health and Nutrition Examination Survey (KNHANES) between 2010 and 2012 were analyzed. The analysis in this study was confined to a total of 1553 respondents over 19 years old who had not gone through menopause and had no missing values for the reproductive factors and outcome variables. A community periodontal index was greater than or equal to code 3 was used to define periodontal treatment needs.

The risk of periodontal treatment needs tended to increase in the presence of menstrual cycle irregularity after adjustment for potential confounders (*P* for trend in the odds ratios = .0481 in model 1; 0.0613 in model 2; 0.0369 in model 3; 0.0456 in model 4). The number of natural teeth of 28 did not reach statistically significant differences (*P* for trend in the odds ratios = 0.2204 in model 1; 0.2373 in model 2; 0.2814 in model 3; 0.2609 in model 4).

Menstrual cycle irregularity was positively associated with the risk of periodontal treatment needs in Korean women before menopause. However, there was no significant association between tooth loss and menstrual cycle irregularity. Menstrual cycle irregularity may be considered to be a potential risk indicator for periodontal treatment needs in Korean women before menopause.

## INTRODUCTION

Menstrual cycles can be important indicators of health and fertility because they reflect basic physiology, and irregular cycles may be indicative of underlying endocrine disorders.^1^ Variability in menstrual cycle length among females is due to the varying number of days required for follicular growth and development in the follicular phase.^2^ Researchers have suggested that menstrual cycle length is a noninvasive clinical marker of reproductive function.^3^ Regular menses require normal plasma estrogen concentrations during the menstrual cycle, and menses become irregular or absent as plasma estrogen values decrease toward postmenopausal concentrations^4^; irregular menses generally reflect hypoestrogenic conditions.^4^

The association between menstrual cycle irregularities and system disease has been evaluated in previous studies. A previous study showed that very irregular menstrual cycles were associated with an increased risk of rheumatoid arthritis.^5^ Women with long or highly irregular menstrual cycles have a significantly increased risk for developing type 2 diabetes mellitus.^6^ Moreover, menstrual cycle length has also been investigated as a predictor of health outcomes including breast cancer and cardiovascular disease risk factors.^3^

The present study aimed to evaluate the relationship between menstrual irregularity and periodontal disease or tooth loss in women before menopause using nationally representative data. To the author's knowledge, this is the first study to elucidate the effect of menstrual irregularity on periodontal disease using nationally representative data.

## METHODS

### Survey and Subjects

This study used data from the Korean National Health and Nutrition Examination Survey (KNHANES), which was conducted between 2010 and 2012 by the Division of Chronic Disease Surveillance under the Korean Centers for Disease Control and Prevention and the Korean Ministry of Health and Welfare.^7^ The KNHANES is a nationwide survey of noninstitutionalized civilians that uses a stratified and multistage probability sampling design with a rolling survey sampling model. The sampling units were based on the population and housing consensus from the National Census Registry in Korea, which includes age, gender, and geographic area. The sample weights were used to calculate all statistics of this survey. Sample weights were created, which considered survey nonresponse, complex survey design, and poststratification to represent the Korean population with sample participants.

Initially, a total of 8058 individuals were candidates in the KNHANES survey. The analysis in this study was confined to a total of 1553 respondents over 19 years old who had not gone through menopause and had no missing values for the reproductive factors and outcome variables. Data regarding reproductive factors were collected by asking the participants to recall the duration of the menstrual cycle, the use of hormone replacement therapy, and the use of oral contraceptives. Menstrual cycle characteristics were categorized as regular, irregular (once within 3 months) and duration longer than 3 months. All participants in the survey signed an informed consent form before participation. This survey was reviewed and approved by the Institutional Review Board of the Korean Centers for Disease Control and Prevention.

### Sociodemographic and Lifestyle Variables

All participants were asked about sociodemographic and lifestyle variables by trained interviewers. Education level was categorized into 2 groups using the criterion of high school graduate or higher. Monthly household income was divided into quartiles after adjusting for the number of family members. The first lowest quartile included households with a monthly income <1092.4 USD. Participants were categorized into 2 groups using the criterion of alcohol consumed within 1 month from the interview.^8^ Smoking status was categorized into 2 groups in accordance with respondents’ answers on the self-report questionnaire: current smoker or not. Based on responses to the modified form of the International Physical Activity Questionnaire for Koreans, individuals were regarded as regular physical exercisers if they performed moderate exercise more than 5 times per week for over 30 minutes per session or performed vigorous exercise more than 3 times per week for over 20 minutes per session.^9^ Sleep duration and recognition of stress were self-reported.

### Anthropometric and Biochemical Measurements

Anthropometric measurements were performed by trained staff members. Body weight and height were measured to the nearest 0.1 kg and 0.1 cm, respectively, with participants in light indoor clothing without shoes. Body mass index was calculated as body weight (kg) divided by the squared height (m^2^). Waist circumference was measured at the narrowest point between the lower border of the rib cage and the iliac crest in a standing position.^10,11^

A standard mercury sphygmomanometer (Baumanometer; W.A. Baum Co., Inc., Copiague, NY) was used for blood pressure measurement. Systolic blood pressure and diastolic blood pressure were measured twice at 5-minute intervals, and the average values were used for the analysis. To measure concentrations of serum fasting plasma glucose, total cholesterol, triglycerides, and high-density lipoprotein–cholesterol, a blood sample was collected from the antecubital vein of each participant after fasting for >8 hours. Blood samples were analyzed within 24 hours of transportation. Levels of serum fasting plasma glucose, total cholesterol, triglycerides, and high-density lipoprotein–cholesterol were measured with a Hitachi Automatic Analyzer 7600 (Hitachi, Tokyo, Japan) by enzymatic methods using commercially available kits (Daiichi, Tokyo, Japan).^12^ HbA1c was measured by high-performance liquid chromatography (HLC-723G7, Tosoh, Japan). Serum 25-hydroxyvitamin D levels were measured using a gamma counter (1470 Wizard; PerkinElmer, Wallac, Turku, Finland) by radioimmunoassay (RIA) using a 25-hydroxyvitamin D ^125^I RIA kit (DiaSorin, Stillwater, MN).

Metabolic syndrome was defined according to the American Heart Association/National Heart, Lung, and Blood Institute Scientific Statement criteria for Asians.^13^ According to this statement, 3 or more of the following criteria must be fulfilled to be diagnosed with metabolic syndrome: waist circumference ≥90 cm in men and ≥80 cm in women; fasting triglycerides ≥150 mg/dL or use of lipid-lowering medication; high-density lipoprotein–cholesterol <40 mg/dL in men and <50 mg/dL in women or use of medication; blood pressure ≥130/85 mm Hg or use of antihypertensive medication; and fasting blood glucose ≥100 mg/dL or current use of antidiabetes medication.

### Oral Health Behaviors, Periodontal Disease, and Number of Natural Teeth

The time of day when participants brushed their teeth and used secondary oral products was recorded as oral health behaviors.^14^ We calculated the frequency of daily tooth brushing by the total number of times the teeth were brushed per day. Self-reported oral state was categorized into favorable, average, and problematic.

The presence of periodontal disease was evaluated using the World Health Organization community periodontal index (CPI). Periodontal disease was defined if CPI was ≥3. When more than 1 or more site had a >3.5 mm pocket in the index teeth, which are 11, 16, 17, 26, 27, 31, 36, 37, 46, and 47 according to the Federation Dentaire Internationale system, it is indicated a CPI score of code 3.^15^ The mouth was divided into sextants and a CPI probe (PWHO, Osung MND, Seoul, Korea) with a 0.5 mm ball tip was used. When 2 or more teeth, which were not scheduled for extraction were present, a sextant was examined. If no index teeth were qualified for the examination in a sextant, all remaining teeth were examined. The highest value for that sextant was used for the score. An average probing force was approximately 20 g.^15^

Twenty-eight teeth excluding third molars were evaluated for the oral health data.^16^ The natural tooth was considered present if the tooth was permanent tooth or primary tooth. It was considered absent if the permanent dental root fragment present. Individuals were classified into 2 categories: 28 or ≤27.

### Statistical Analyses

All data are presented as mean ± standard error or percentage (standard error). A Chi-square test for categorical variables or an independent *t* test for continuous variables was performed to assess the differences in characteristics according to the presence of periodontal disease or number of natural teeth of 28. A hierarchical multivariable logistic regression analysis was used to evaluate the risk of periodontal disease in relation to menstrual cycle irregularity, and odds ratios and 95% confidence intervals were calculated after adjusting for potential confounders. Model 1 was adjusted for age and body mass index. Variables adjusted in model 1 plus smoking, drinking, exercise, education, and income were adjusted in model 2.

Model 3 was adjusted for the variables adjusted in model 2 plus metabolic syndrome and stress. Model 4 was adjusted for the variables adjusted in model 3 plus the frequency of tooth brushing, use of secondary oral products, dental checkup, hormone replacement therapy, and oral contraceptive. Statistical analyses were performed by using the survey procedure of SAS (Version 9.2, SAS Institute, Cary, NC,) to account for the complex sampling design. Two-sided *P* values of <0.05 were considered an indicator of statistical significance.

## RESULTS

Table 1 describes the baseline characteristics of the study individuals according to the presence of periodontal treatment needs and the number of natural teeth (28 or 27 or less). The mean age, body mass index, waist circumference, menstrual cycle irregularity, systolic blood pressure, diastolic blood pressure, glucose, HbA1c, serum 25-hydroxyvitamin D, cholesterol, low-density lipoprotein, triglyceride, and low income were significantly higher in participants with periodontal treatment needs. High-density lipoprotein and high education were significantly lower in participants with periodontal treatment needs. Similar trends were seen with the number of natural teeth 27 or lower when compared with the participants with 28 natural teeth.

**TABLE 1:**
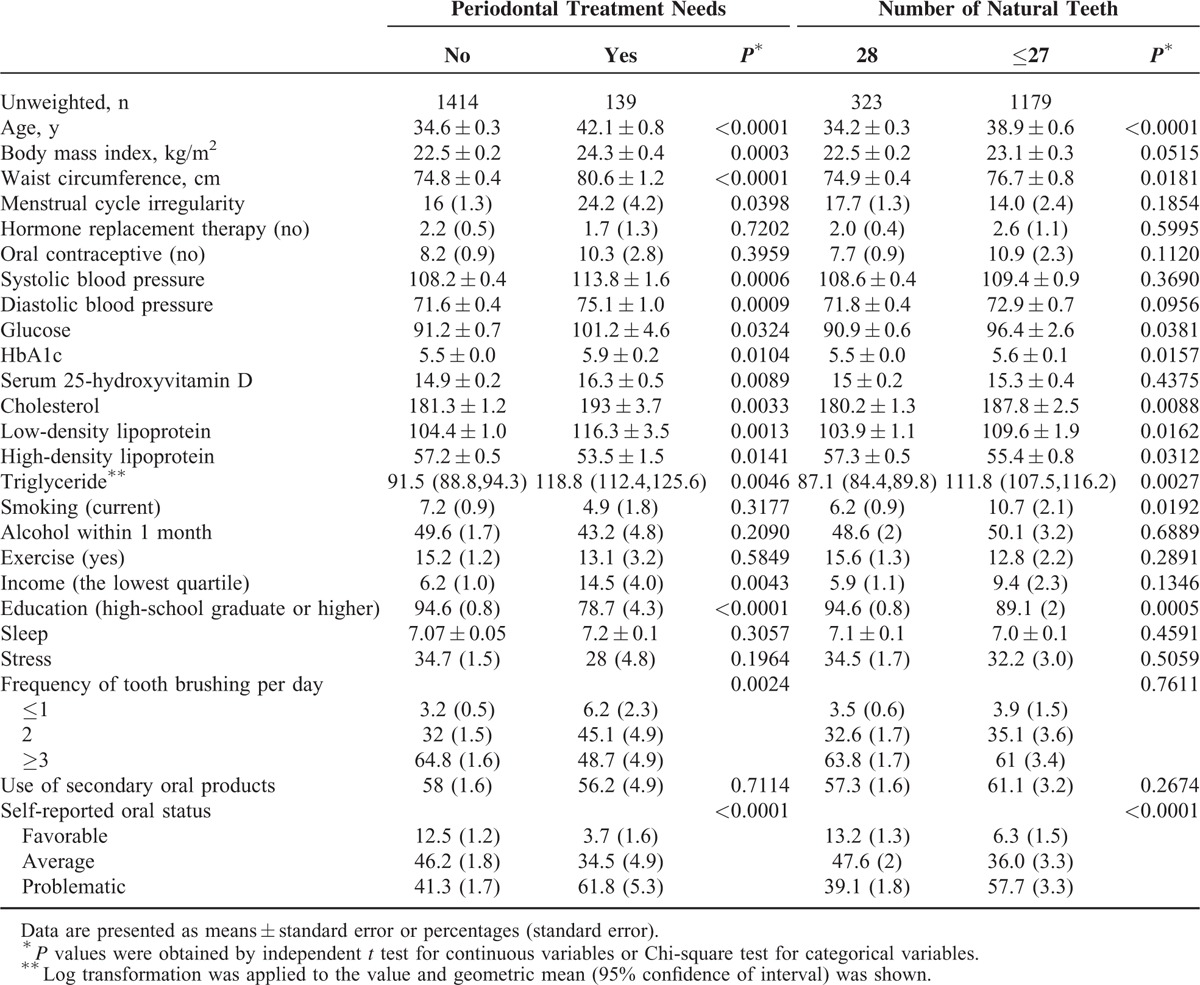
Baseline Characteristics of Study Individuals According to Periodontal Treatment Needs and the Number of Natural Teeth

Table 2 shows the effects of body mass index or waist circumference on menstrual cycle irregularity, periodontal treatment needs, and number of natural teeth of 28. Menstrual cycle irregularity was more prevalent in individuals with higher body mass index or waist circumference (*P* values for trend of 0.0240 and 0.1391, respectively). Periodontal disease was more prevalent in individuals with higher body mass index or waist circumference (*P*-value for trend of <0.0001).

**TABLE 2:**
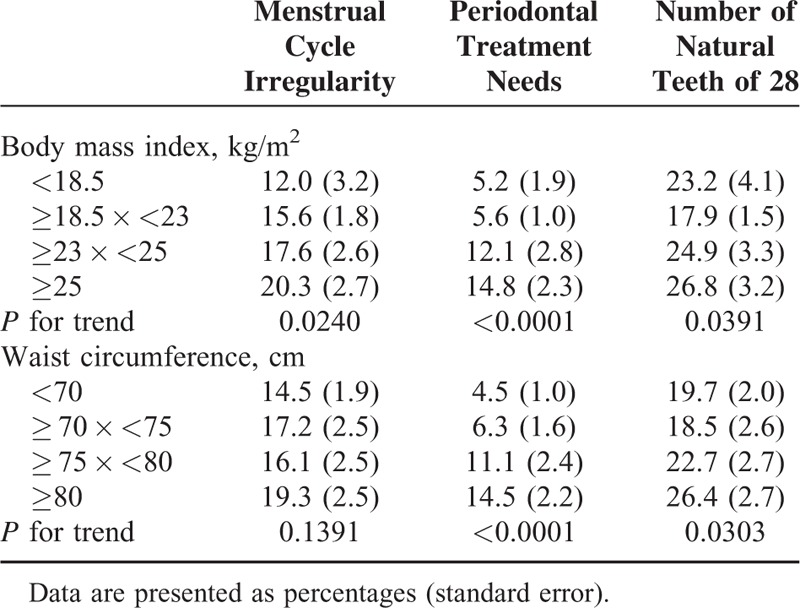
The Effects of Body Mass Index or Waist Circumference on Menstrual Cycle Irregularity, Periodontal Treatment Needs and Number of Natural Teeth of 28

**FIGURE 1:**
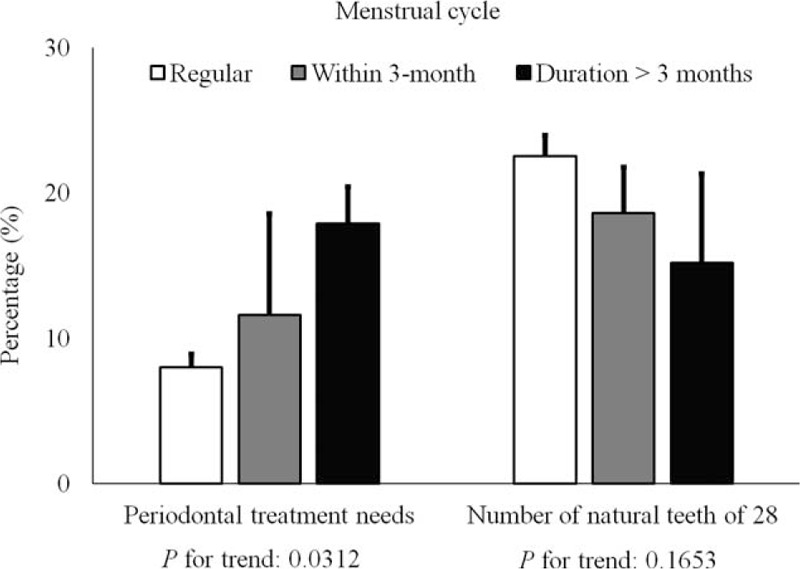
The percentage of individuals with periodontal treatment needs (*P* for trend: 0.0312) or 28 natural teeth (*P* for trend: 0.1653) according to menstrual cycle.

Figure 1 shows the percentage of individuals with periodontal treatment needs or the 28 natural teeth regarding the menstrual cycle. *P* values for trend of the periodontal treatment needs and number of natural teeth of 28 were 0.0312 and 0.1653, respectively. The percentage of individuals with periodontal treatment needs with regular menstrual cycles was 8.0 ± 0.9%. The percentage of individuals with periodontal treatment needs with irregular menstrual cycles of once in 3 months and duration longer than 3 months were 17.9 ± 7.0% and 18.6 ± 3.2%, respectively.

Table 3 shows the adjusted odds ratios and their 95% confidence intervals from multivariate logistic regression analyses for individuals with the periodontal treatment needs and the number of natural teeth of 28. Adjusted odds ratios and their 95% confidence intervals of the individuals with periodontal treatment needs were 1.764 (1.011–3.076) after adjustment with age, body mass index, smoking, drinking, exercise, education, income, metabolic syndrome, stress, the frequency of tooth brushing, use of secondary oral products, dental checkup, hormone replacement therapy, and oral contraceptive (model 4).

**TABLE 3:**
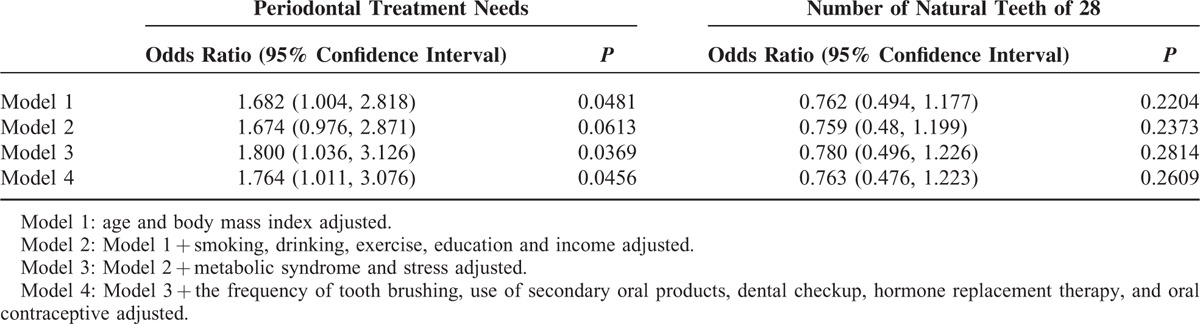
Adjusted Odds Ratio and 95% Confidence Interval of the Individuals With Periodontal Treatment Needs or the Number of Natural Teeth of 28 in Multivariate Logistic Regression Model in the Presence of Menstrual Cycle Irregularity

## DISCUSSION

This study aimed to identify associations between the periodontal disease in women before menopause and menstrual cycle irregularity. The result showed that an increased risk of periodontal disease was associated with menstrual cycle irregularity in women before menopause with statistical significance.

Assessment of menstrual cycle characteristics differed among different studies.^2,4,17^ The mechanism underlying the association between periodontal disease and menstrual cycle irregularity has not been fully revealed. Participants identified their menses as regular, irregular, or amenorrheic by evaluating their present and previous 12-month menstrual history by using one of the following 3 categories: regular (11–13 menses/year), irregular (3–10 menses/year), and amenorrheic (≤2 menses/year).^4^ The periods were defined as regular if the overall range was within 20 to 40 days in another study.^2^ Another report described self-reported menstrual cycle characteristics as short (≤25 days), normal (26–34 days), or long (≥35 days), and cycles were defined as irregular if there were ≥15 days between the longest and shortest cycle in the previous 12 months.^17^ In this study, menstrual cycle characteristics were categorized as regular, irregular (once within 3 months) and duration longer than 3 months.

It was suggested that one of the principal factors for cycle regularity is body weight.^2^ A previous study revealed that obese women are more likely to experience menstrual cycle irregularities, including amenorrhea and oligomenorrhea, than nonobese women.^18^ A total of 26% of those who had body mass index of 30 or higher had irregular menstrual cycles compared with 14% of those with a body mass index in the range 20 to 24.9. Similar findings were observed when obesity was defined as a waist circumference of ≥88 cm.^17^ Centrally distributed body fat was suggested to be more strongly associated with menstrual abnormalities and adverse hormonal profiles than measures of peripheral body fat or overall adiposity such as body mass index.^17^ The association between obesity and periodontal disease was proved previously,^19–21^ and this may explain the association between menstrual cycle irregularity and periodontal treatment needs.

Previous studies showed that high androgen levels have been associated with menstrual irregularities in clinical populations.^1,17^ Increased androgen levels have been associated with central and peripheral adiposity.^17^ Moreover, elevated levels of androgen could affect the cell response to an inflammatory challenge by downregulation of antiinflammatory cytokine of IL-6 production.^22^

There may be some limitations that should be considered. First of all, we cannot be certain of the causal direction of the associations observed due to the study's cross-sectional design; a longitudinal study with repeated measures of the presence of periodontal treatment needs and menstrual characteristics would be desirable in future.^17^ Another limitation of this study is that participants’ self-reported cycle characteristics retrospectively rather than using menstrual diaries,^1,6,23^ and the exact length of each was not reported in this study. Considerable measurement error was noted in self-reported cycle length, and agreement between observed and reported cycle length was moderate.^4^ However, it should be emphasized that the KNHANES data are highly reliable because they were obtained from a nationwide, population-based, and representative sample and because the analysis used sample weights and adjustments for the complex sample design of the survey.^24,25^

This study investigated the relationship between menstrual cycle irregularity and periodontal treatment needs or the number of natural teeth and yielded several important findings, the most significant being the identification of an association between periodontal treatment needs and menstrual cycle irregularity. However, there was no significant association between tooth loss and menstrual cycle irregularity. Menstrual cycle irregularity was discovered to be a potential risk indicator for periodontal disease in Korean women before menopause.
